# *In Vitro* Free Radical Scavenging Properties and Anti-Inflammatory Activity of *Ilex paraguariensis* (Maté) and the Ability of Its Major Chemical Markers to Inhibit the Production of Proinflammatory Mediators

**DOI:** 10.1155/2021/7688153

**Published:** 2021-11-01

**Authors:** Ingrid Vicente Farias, Eduarda Fratoni, Lais Cristina Theindl, Angela Machado de Campos, Eduardo Monguilhott Dalmarco, Flavio Henrique Reginatto

**Affiliations:** Programa de Pós-Graduação em Farmácia, Centro de Ciências da Saúde, Universidade Federal de Santa Catarina, 88040-900 Florianópolis, Brazil

## Abstract

*Ilex paraguariensis* A. St. Hil. (Aquifoliaceae), popularly known as “*yerba mate*,” has great economic and social significance for the population of Southern Latin America. This study was conducted (1) to investigate the phytochemical composition of four different standardized extracts, (2) to investigate its free radical scavenging properties, and (3) to investigate the anti-inflammatory action of *I. paraguariensis* and its major chemical markers. The chemical profile was achieved by Folin-Ciocalteu, by LC/DAD, and by LC/MS assays, while the antioxidant and anti-inflammatory properties were investigated, respectively, by DPPH assay and by inhibition of nitric oxide (Griess reaction) and TNF-*α* (ELISA). Our results demonstrated that the IA (aqueous infusion extract) showed higher amounts of total phenolic contents (266.62 ± 10.85 mg CAE·g^−1^ DE), the highest amounts of all six chemical markers (theobromine, 5-*O*-caffeoylquinic acid, 4-*O*-caffeoylquinic acid, 3-*O*-caffeoylquinic acid, caffeine, and rutin), and stronger antioxidant activity (EC_50_ = 54.4 ± 5.14 *μ*g · mL^−1^). The IA extract also showed the lowest inhibition of NOx secretion (50.10 ± 8.97%) as well as inhibition of TNF-*α* (83.33 ± 4.01%). Regarding the chemical markers, all compounds showed strong inhibition of NOx secretion, especially theobromine, which was 200x more potent than dexamethasone. Furthermore, TNF-*α* secretion was also significantly decreased by THEO at 0.033 *μ*M (22.15 ± 6.49%), NCA at 1.97 *μ*M (27.46 ± 3.98%), CCA at 0.35 *μ*M (39.76 ± 5.73%), CGA at 0.56 *μ*M (23.58 ± 5.79%), CAF at 0.52 *μ*M (26.45 ± 5.34%), and RUT at 0.16 *μ*M (40.18 ± 3.70%). Our results suggest that *I. paraguariensis* and its major chemical markers have strong free radical scavenging properties as well as showed important anti-inflammatory activity and that these compounds in a plant extract may work based on several different mechanisms synergistically, resulting in moderating the immune system.

## 1. Introduction

Inflammation usually occurs as a protective event to rid the organism of harmful stimuli. Inflammation may also happen in response to processes such as tissue injury, cell death, cancer, ischemia, and degeneration, leading to the activation of intracellular pathways and triggering the kinase cascades and nuclear transcription factors, such as MAPKs and NF-*κ*B [1, 2]. Although the development of anti-inflammatory drugs has improved the treatment of several diseases, other inflammatory pathologies such as asthma, atherosclerosis, and sepsis have not shown adequate response to current medications. Moreover, the chronic use of these drugs is reported to cause severe adverse effects, including gastrointestinal, cardiovascular, and renal abnormalities [3, 4]. Free radicals are also related to diseases associated with elevated inflammatory signaling. Atherosclerosis, diabetes, and Alzheimer's are related with an altered redox balance. Therefore, the investigation of new anti-inflammatory and free radical scavenging agents remains relevant [5, 6].

The in vitro models represent the beginning of the rational research, this complex and intricate process needs a fast and reliable screening to follow to the next level of pharmacological research, and the model using RAW 264.7 cells is widespread in research laboratories worldwide due to its versatility and reproducibility in the results [7, 8]. Moreover, the most common inflammatory biomarkers evaluated in this model are the nitric oxide (NOx) and tumoral necrosis factor alpha (TNF-*α*); both are produced immediately after initial action of the phlogistic agent (LPS) that induced the activation of nuclear transcription factor-kB (NF-*κ*B) [9, 10].

In this context, natural products can be an effective alternative in the treatment of inflammation, since medicinal plants are used worldwide as an efficient treatment of many diseases, including inflammation. Also, natural products have produced significant numbers of agents used as drugs or drug leads in recent decades [11].

*Ilex paraguariensis* A. St. Hil. (Aquifoliaceae) or “*yerba mate*” has great economic and social significance for the population of Southern Latin America in view of the high consumption of mate, terere, or mate-tea [12]. Yerba mate is also used in popular medicine for liver protection, inflammation, weight reduction, and cholesterol [13]; pharmacological properties all of which are linked to the presence of methylxanthines [14], saponins [15], and phenolic compounds [12, 16].

There are several *in vitro* and *in vivo* pharmacological studies that show modulation of lipid metabolism and antioxidant, antidiabetic [12, 17, 18], antiobesity [13], and anti-inflammatory properties [18–20], as well as central nervous system stimulant and neuroprotective effects [21] for *I. paraguariensis* extracts. Regarding the anti-inflammatory properties, there is one clinical study that showed this activity for *I. paraguariensis* extracts [22] and few works that investigated the anti-inflammatory properties of isolated compounds from *I. paraguariensis* [19]. However, some of these works were conducted using no standardized extract preparations, which may have an impact on the physicochemical stability of the extracts as well as on the reproduction of the results [23, 24].

Phytopharmaceutical products can represent an enormous challenge in the search for new anti-inflammatory agents with a high level of uniformity, reproducibility, and stability [23, 24]. Moreover, polyphenol compounds, the major phytochemicals present in *I. paraguariensis* preparations, can regulate immunity by interfering with immune cell regulation and proinflammatory cytokines' synthesis, inactivate NF-*κ*B (nuclear factor kappa-light-chain-enhancer of activated B cells), and modulate the mitogen-activated protein kinase (MAPK) and arachidonic acid pathways [25]. Therefore, we aimed in this work to evaluate the *in vitro* free radical scavenging and the ability of different standardized extracts of *I. paraguariensis* as well as its major chemical markers to inhibit the production of proinflammatory mediators.

## 2. Materials and Methods

### 2.1. Plant Material

The plant material was purchased from the local market in the city of Florianópolis, in the state of Santa Catarina, Brazil, in 2018. The plant material was botanically characterized in accordance with the Argentine Pharmacopeia [26] and the Brazilian Pharmacopeia [27].

### 2.2. Preparation of Extracts

The extracts were prepared using two different extraction methods: infusion and turboextraction as previously described [28]. In both techniques, the extracts were produced using a raw material : solvent ratio of 5% (*w*/*v*). Crushed leaves of *I paraguariensis* were subjected to the infusion or turboextraction with water or hydroethanolic solution (20%) for 30 min. The extracts prepared by turboextraction were prepared using an Ultra-TURRAX® (model T25, IKA®, Germany; catalogue number 0003725032), with a S25N-10G stirring stem (IKA®, Germany; catalogue number 0000594000), at a stirring speed of 9500 rpm, for 5 minutes, with water or a hydroethanolic solution (20%). All four extracts were then filtered and dried using a spray dryer. The spray dryer conditions used were Mini Spray Dryer B-290 (Buchi®; catalogue number 044699) with a two-component nozzle and current flow under the following operating conditions: inlet temperature of 160°C, outlet temperature 100°C, 10% pump flow (3 mL/min), 100% aspirator, and atomizer diameter 0.7 mm. The spray-dried extracts were identified as IA (aqueous infusion), IE (hydroethanolic infusion), TA (aqueous turboextraction), and TE (hydroethanolic turboextraction). All procedures were conducted in triplicate.

### 2.3. Chemical Characterization

#### 2.3.1. Total Phenolic Content (TPC)

Total phenolic content was determined by the Folin-Ciocalteu (FC, Sigma-Aldrich®; catalogue number F9252) assay [29, 30]. The samples were analyzed in triplicate, and the results were expressed as milligrams of chlorogenic acid equivalents per gram of dry extract (mg CAE·g^−1^ DE). An analytical curve was plotted with chlorogenic acid in the range of 500-15 *μ*g·mL^−1^ (*R*^2^ = 0.999).

#### 2.3.2. Chemical Characterization by UPLC/PDA Analysis

The chemical markers were quantified in order to evaluate the extraction process. This quantification was performed using an UPLC (Waters® Acquity™ UPLC™ H-Class; catalogue number 186015018) coupled to photodiode array detector (PDA, Waters®; catalogue number 186015033). The separation was achieved on reversed phase column Phenomenex® brand Kinetex model (C18, 2.1 × 150 mm) with a particle size of 2.6 *μ*m (catalogue number PN 00A-4461-AN).

The mobile phase was a gradient form combining solvent A (H_2_O, 0.1% formic acid, Merck®; catalogue number 100264) and solvent B (MeOH, Merck®; catalogue number 106007), programmed as follows: 0-2 min, linear change from A : B (85 : 15) to A : B (75 : 25); 2-4 min, linear change from A : B (75 : 25) to A : B (40 : 60); 4-9 min, linear change from A : B (40 : 60) to A : B (30 : 70); 9-10 min, linear change from A : B (30 : 70) to A : B (85 : 15); and 10-12 min isocratic A : B (85 : 15), with a flow rate of 0.9 mL min^−1^.

The following chemical markers (all from Sigma-Aldrich®) were used for the quantification: theobromine (THEO, catalogue number T4500), 5-*O*-caffeoylquinic acid (neochlorogenic acid (NCA), catalogue number 94419), 4-*O*-caffeoylquinic acid (cryptochlorogenic acid (CCA), catalogue number 65969), 3-*O*-caffeoylquinic acid (chlorogenic acid (CGA), catalogue number 3818), caffeine ((CAF), catalogue number BP766), and rutin ((RUT), catalogue number R5143). The wavelengths used for detection were 280 nm for methylxanthines and 320 nm for phenolic acids and flavonoids.

#### 2.3.3. Chemical Characterization by LC/MS Analysis

The LC/MS analysis was performed according Feltrin et al. [31]. Briefly, the compounds were identified by high-performance liquid chromatography (Waters® Acquity™ UPLC™; catalogue number 186015018) coupled to a photodiode array detector (PDA, Waters®; catalogue number 186015033) and a high-resolution mass spectrometer (Xevo®G2 QTof, WATERS® model; catalogue number 186006532) equipped with a source of electrospray ionization (ESI) operating in positive and negative mode. The mobile phase was consisted by a gradient of 0.1% of aqueous formic acid (A) and methanol (B) at constant flow rate (0.5 mL/min). This gradient was programmed as follows: initial condition 15-85% (B-A), a linear gradient from 15 to 25% (B) for 2 min, 25-60% (B) for 2-4 min, 60-70% (B) for 4-9 min, and 70-15% (B) for 9-10 min. The mass spectrometer parameters were set as follows: for ESI−, capillary voltage of 2.5 kV, source block temperature of 150°C; desolvation temperature of 500°C, nebulizer nitrogen flow rate of 150 L/h, and desolvation nitrogen gas flow of 1000 L/h; for ESI+, capillary voltage of 3.5 kV, source block temperature of 90°C, desolvation temperature of 400°C, nebulizer nitrogen flow rate of 30 L/h, and desolvation nitrogen gas flow of 900 L/h were set. MS/MS analyses were performed using a collision energy ramp 10-30 eV.

### 2.4. Screening of Biological Activities

#### 2.4.1. Radical Scavenging Assay—DPPH Assay

The radical scavenging assay of the four samples (IA, IE, TA, and, TE) and chemical markers (THEO, NCA, CCA, CGA, CAF, and RUT) was performed by the DPPH (2,2-diphenyl-1-picrylhydrazyl, Sigma-Aldrich®; catalogue number D9132) assay, described by Brand-Williams et al. [32] and modified by Zhu et al. [33]. The analysis was performed in triplicate, and the concentration of each sample that reduced 50% of the DPPH concentration (EC_50_) was expressed as *μ*g·mL^−1^ for the extracts and *μ*M for the chemical markers.

#### 2.4.2. In Vitro Cytotoxicity Assay

The cytotoxicity of *I. paraguariensis* extracts on RAW 264.7 from American Type Culture Collection (ATCC® TIB-71™) was evaluated using 3-(4,5-dimethylthiazol-2-yl)-2,5-diphenyltetrazolium bromide (MTT, Sigma-Aldrich®; catalogue number M5655) [34]. Briefly, the cells were grown in appropriate plastic bottles with Dulbecco's Modified Eagle's Minimum Essential Medium (DMEM, Gibco®; catalogue number 11965-092), supplemented with 10% of inactive fetal bovine serum (FBS, Gibco®; catalogue number 12657-029) and 100 U/mL penicillin and 100 *μ*g/mL streptomycin (Pen/Strep, Sigma-Aldrich®; catalogue number P4333), in an incubator humidified at 37°C with 5% CO_2_ emissions. Before the experiments, the number of viable cells was determined by the trypan blue exclusion method, performing the counts in a Neubauer chamber. Afterwards, the cells were distributed into 96-well culture microplates (1 × 10^4^ cells/well) and incubated with the extracts at different concentrations (3, 10, 30, 100, 300, and 1000 *μ*g·mL^−1^). Dimethyl sulfoxide (DMSO, Sigma-Aldrich®; catalogue number 472301) was used to solubilize the samples at a maximum concentration of 1% per well/treatment, at which concentration at which there is no cytotoxicity to the cells. After the treatment period (24 h), the medium was removed and 100 *μ*L of a MTT solution was added to 0.5 mg·mL^−1^ in culture medium and incubated for 2 hours. After this period, the medium was removed, the formazan precipitate was dissolved in 100 *μ*L DMSO/well, and the absorbance was measured at 540 nm (ELISA Reader MR96A, Mindray®; catalogue number 104-20-62564). The optical density obtained in the control group—untreated cells (incubated with growth medium only)—was regarded as 100% viable cells. The cytotoxic concentration (CC_10_), which is the concentration required to reduce cell viability by 90%, was calculated through a Hill concentration-response curve using the software Prism 6.0 (GraphPad Software, La Jolla, CA, USA).

#### 2.4.3. In Vitro Inflammation Assay

To induce the RAW 264.7 macrophages to an inflammatory condition, cells were cultured in a 96-well plate (2 × 10^5^ cell/well) for 48 h until macrophage adherence and confluence (at 37°C in a 5% CO_2_ humidified atmosphere). When adherence was adequate (±90%), cells were designated to different groups (*n* = 3/group), as follows: (a) blank control (B, uninflamed cells), cells pretreated with vehicle; (b) negative control (LPS, inflamed cells) (LPS, Sigma-Aldrich®; catalogue number L2630), also cells pretreated with vehicle; (c) positive control (dexamethasone (DEX), standard treatment) (DEX, Sigma-Aldrich®; catalogue number D4902) cells pretreated with DEX (7 *μ*M), and (d) experimental groups (extract and compound treatments). After 1 h, the cells were stimulated with LPS (1 *μ*g/mL) for 24 h, and then, the supernatants were collected for further investigations upon nitric oxide metabolite (NO_x_) production.

#### 2.4.4. NOx Measurement

Nitrite accumulation in the culture supernatants was measured as an indicator of NO production based on the Griess reaction [35]. To evaluate which extraction procedure had the highest anti-inflammatory properties, the nitrite production was evaluated in the following groups: (a) IA, (b) IE, (c) TA, and (d) TE. Furthermore, in order to evaluate the importance of each compound on anti-inflammatory profile of the IA extract, we evaluated the effect of these compounds on the NOx levels at the concentration present in the original extract (IA extract at 3 *μ*g·mL^−1^). The groups for these experiments were as follows: (a) THEO at 0.033 *μ*M, (b) NCA at 1.97 *μ*M, (c) CCA at 0.35 *μ*M, (d) CGA at 0.56 *μ*M, (e) CAF at 0.52 *μ*M, and (f) RUT at 0.16 *μ*M. In all experiments, 100 *μ*L of cell culture medium was collected 24 h after LPS stimulation (1 *μ*g·mL^−1^), mixed with an equal volume of Griess reagent and incubated at room temperature for 10 min. Absorbance at 540 nm was measured with interpolation from the nitrite standard curve (0–100 *μ*M). The nitrite production was determined, and the results were expressed in *μ*M. All experiments were performed in triplicate.

#### 2.4.5. Proinflammatory Cytokine Measurement (TNF-*α*)

The levels of TNF-*α* in the cell supernatant were quantified for all extracts (IA, IE, TA, and TE) as well as for each compound at the concentration present in the IA extract at 3 *μ*g·mL^−1^. The groups for these experiments were as follows: (a) THEO at 0.033 *μ*M, (b) NCA at 1.97 *μ*M, (c) CCA at 0.35 *μ*M, (d) CGA at 0.56 *μ*M, (e) CAF at 0.52 *μ*M, and (f) RUT at 0.16 *μ*M. The concentrations of TNF-*α* were determined using a commercially available enzyme-linked immunosorbent assay kit (Mouse TNF-*α*, PeproTech®, Rocky Hill, NJ, USA; catalogue number 900-T54) according to the manufacturer's instructions. Cytokine level was estimated by interpolation from the standard curve, and the results are expressed in pg/mL.

### 2.5. Statistical Analysis

The data were presented as mean ± standard deviation. Statistical analyses were performed using GraphPad Prism 6 software®. Data were submitted to analysis of variance (ANOVA) followed by Tukey post hoc test (*p* < 0.05).

## 3. Results

### 3.1. Total Phenolic Content (TPC)

**Figure 1:**
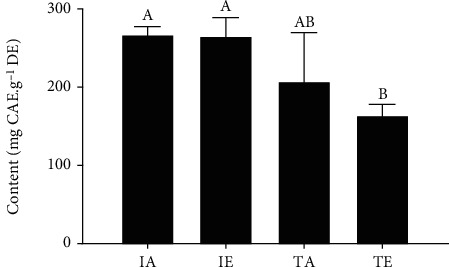
Total phenolic content by Folin-Ciocalteu assay. Different letters (A, B) indicate significant difference (ANOVA, followed by Tukey test; *p* < 0.05). IA: aqueous infusion; IE: hydroethanolic infusion; TA: aqueous turboextraction; TE: hydroethanolic turboextraction.

The TPC values are shown in Figure 1. The analysis showed that all extracts have similar TPC, except for TES extract, which showed lower TPC (*p* < 0.05) than all the other preparations.

### 3.2. UPLC-PDA Analysis

**Figure 2:**
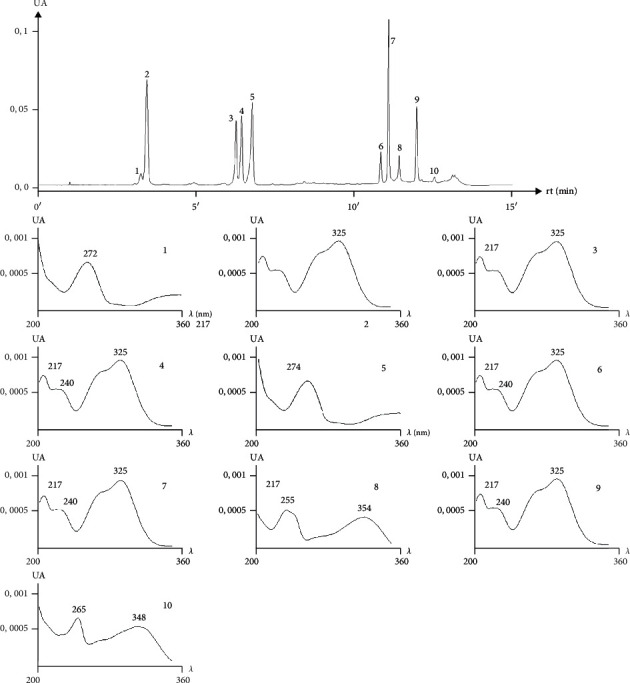
LC-UV chromatographic profile at 270 nm and UV profile of *Ilex paraguariensis* aqueous extract: (1) theobromine; (2) neochlorogenic acid; (3) cryptochlorogenic acid; (4) chlorogenic acid; (5) caffeine; (6) di-O-caffeoylquinic acid derivatives; (7) di-O-caffeoylquinic acid derivatives; (8) rutin; (9) di-O-caffeoylquinic acid derivatives; (10) not identified.

The extracts showed the same fingerprint, but differences in the amounts of chemical markers were detected. Moreover, the UV profiles observed for these compounds were in agreement with those described in the literature [19]. The typical qualitative chromatographic profile of IA and the UV profile of its chemical markers are shown in Figure 2. The quantification of chemical markers is presented in Table 1. The IA was the extract that showed the higher amounts of all seven chemical markers, in a single extract.

### 3.3. UPLC-MS Analysis

**Figure 3:**
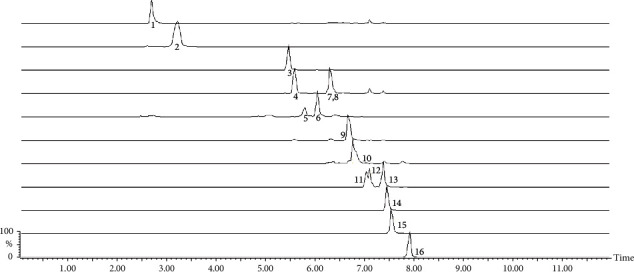
LC-MS chromatographic profile of *Ilex paraguariensis* extract.

The LC/MS identified the presence of quinic acid, citric acid, theobromine, caffeine, 5-*O*-caffeoylquinic acid, 4-*O*-caffeoylquinic acid, 3-*O*-caffeoylquinic acid, caffeoylglucose, feruloylquinic acid, 4,5-di-O-caffeoylquinic acid, 3,5-di-O-caffeoylquinic acid, 3,4-di-O-caffeoylquinic acid, rutin, quercetin-glycoside, and kaempferol-rhamnoglucoside in all four *I. paraguariensis* extracts presented in Figure 3 and Table 2.

### 3.4. Screening of Biological Activities

#### 3.4.1. Radical Scavenging Assays—DPPH Assay

**Figure 4:**
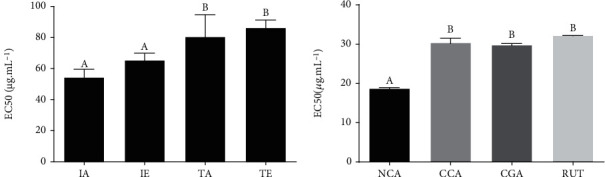
Radical scavenging by DPPH assay expressed in EC_50_: (a) extracts and (b) compounds. Different letters (A, B) indicate significant difference (ANOVA, followed by Tukey test; *p* < 0.05). Note: IA: aqueous infusion; IE: hydroethanolic infusion; TA: aqueous turboextraction; TE: hydroethanolic turboextraction; NCA: neochlorogenic acid; CCA: cryptochlorogenic acid; CGA: chlorogenic acid; RUT: rutin.

In the present study, the DPPH assay was used to evaluate the free radical scavenging of extracts and the major substances identified in the extracts. The EC_50_ ranged from 86.2 ± 5.0 to 54.4 ± 5.1 *μ*g · mL^−1^ for all extracts tested, although the IAS extract showed the lowest EC_50_ (54.4 ± 5.1 *μ*g · mL^−1^). The results are shown in Figure 4(a). The EC_50_ of each chemical marker were also investigated; the results are shown in Figure 4(b). NCA, CCA, CGA, and RUT present significant activity, showing an EC_50_ of 18.72 ± 0.2 *μ*M, 30.40 ± 1.13 *μ*M, 29.78 ± 0.43 *μ*M, and 32.18 ± 0.04 *μ*M, respectively. As expected, caffeine and theobromine did not show free radical scavenging by the DPPH assay.

#### 3.4.2. Cytotoxicity In Vitro Assay

**Figure 5:**
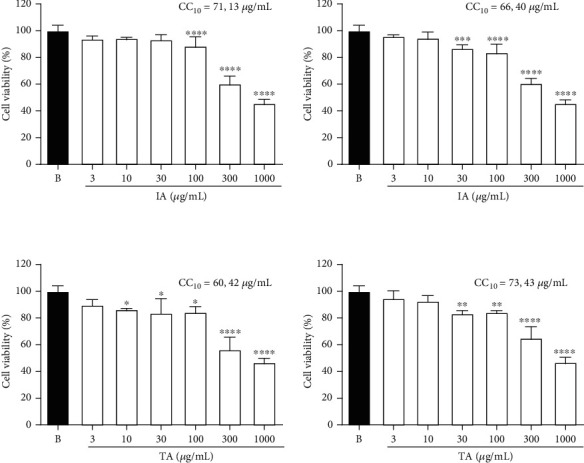
Effect of *Ilex paraguariensis* extracts (IA, IE, TA, and TE) on cytotoxicity of RAW 264.7 cells. B: untreated cells. Results were expressed as mean ± SEM; *n* = 3; ^∗^*p* < 0.1, ^∗∗^*p* < 0.01, ^∗∗∗^*p* < 0.001, and ^∗∗∗∗^*p* < 0.0001 vs. group B. IA: aqueous infusion; IE: ethanolic infusion; TA: aqueous turboextraction; TE: ethanolic turboextraction.

The cytotoxicity effect of *Ilex* extracts on RAW 264.7 macrophages was determined using the MTT assay. The cells were incubated with different concentrations of the extracts (3-1000 *μ*g·mL^−1^). All extracts showed a CC_10_ higher than 50 *μ*g·mL^−1^ (Figure 5). Therefore, to ensure that only nontoxic concentrations of the extract would be used, all subsequent experiments were conducted at concentrations below 50 *μ*g·mL^−1^ (3, 10, and 30 *μ*g·mL^−1^).

#### 3.4.3. Anti-Inflammatory In Vitro Assay

**Figure 6:**
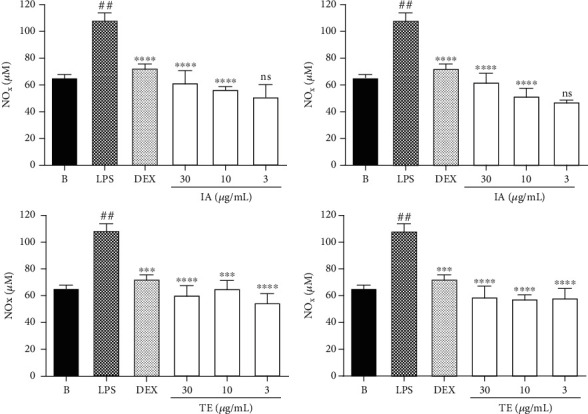
Effects of *Ilex paraguariensis* extracts on NOx level in LPS-treated RAW 264.7 macrophages. B: untreated cells; LPS: cells treated with only lipopolysaccharide (1 *μ*g/mL); DEX: cells pretreated with dexamethasone (7 *μ*M) before LPS administration. Results were expressed as mean ± SEM; *n* = 3; ^∗∗∗^*p* < 0.001 and ^∗∗∗∗^*p* < 0.0001 vs. group LPS; ^##^*p* < 0.01 vs. group B. IA: aqueous infusion; IE: ethanolic infusion; TA: aqueous turboextraction; TE: ethanolic turboextraction.

The anti-inflammatory activity was measured by NOx secretion in LPS-treated macrophages. As expected, LPS increase the levels of NOx secretion, while the anti-inflammatory drug dexamethasone (DEX), at 7 *μ*M, reduced the levels of this proinflammatory mediator release (% inhibition: 29.10 ± 3.05) (*p* < 0.001). NOx secretion was also inhibited by different extracts and ranged from 36.05 to 53.57% (Figure 6). Furthermore, the NOx secretion was significantly diminished by the IA at doses of 30 *μ*g·mL^−1^ (% inhibition: 39.76 ± 8.82) (*p* < 0.001), 10 *μ*g·mL^−1^ (% inhibition: 44.45 ± 1.96) (*p* < 0.0001), and 3 *μ*g·mL^−1^ (% inhibition: 50.10 ± 8.97) (*p* < 0.0001), as well as by IE at doses of 30 *μ*g·mL^−1^ (% inhibition: 39.09 ± 6.24) (*p* < 0.0001), 10 *μ*g·mL^−1^ (% inhibition: 49.23 ± 5.52) (*p* < 0.0001), and 3 *μ*g·mL^−1^ (% inhibition: 53.57 ± 1.25) (*p* < 0.0001).

The extracts prepared by turboextraction also showed significant inhibition of NOx secretion. This activity was detected for TA extract at doses of 30 *μ*g·mL^−1^ (% inhibition: 40.69 ± 6.74) (*p* < 0.0001), 10 *μ*g·mL^−1^ (% inhibition: 36.05 ± 5.90) (*p* < 0.001), and 3 *μ*g·mL^−1^ (% inhibition: 46.33 ± 6.49) (*p* < 0.0001) while the TE extract showed significant inhibition of NOx secretion at doses of 30 *μ*g·mL^−1^ (% inhibition: 42.13 ± 7.82) (*p* < 0.0001), 10 *μ*g·mL^−1^ (% inhibition: 43.58 ± 2.89) (*p* < 0.0001), and 3 *μ*g·mL^−1^ (% inhibition: 42.86 ± 6.83) (*p* < 0.0001) (Figure 5). The IE (53.6%) and IA (50.1%) extracts showed the highest inhibition of NOx secretion at the lower concentration tested (3 *μ*g·mL^−1^).

Considering the results of NOx inhibition by the different *I. paraguariensis* extracts, its free radical scavenging activities, and its high amounts of chemical markers, it is possible to verify that the most effective results were produced when cells were treated with IA extract. Thus, to clarify the putative compounds responsible for the anti-inflammatory effect detected, the evaluation of THEO (theobromine), NCA (neochlorogenic acid), CCA (cryptochlorogenic acid), CGA (chlorogenic acid), CAF (caffeine), and RUT (rutin) on NOx secretion and TNF-*α* level was conducted, using the same concentration as that present in the aqueous extract (IA).

**Figure 7:**
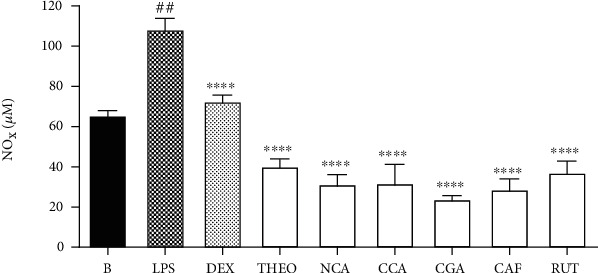
Effects of chemical marker compounds on NOx secretion levels in LPS-treated RAW 264.7 macrophages. B: untreated cells; LPS: cells treated with only lipopolysaccharide (1 *μ*g/mL); DEX: cells pretreated with dexamethasone (7 *μ*M) before LPS administration. Results were expressed as mean ± SEM; *n* = 3; ^##^*p* < 0.0001 vs. group LPS; ^∗∗∗∗^*p* < 0.01 vs. group B. THEO: theobromine; NCA: neochlorogenic acid; CCA: cryptochlorogenic acid; CGA: chlorogenic acid; CAF: caffeine; RUT: rutin.

In these experiments, the NOx secretion was significantly inhibited by all chemical markers present in IA extract when tested separately (Figure 7). The LPS increase the NOx secretion levels, while the dexamethasone (DEX), at 7 *μ*M, reduced the levels of this proinflammatory mediator (% inhibition: 29.10 ± 3.05) (*p* < 0.001). Furthermore, NOx secretion was significantly decreased by THEO tested at 0.033 *μ*M (% inhibition: 60.72 ± 3.61) (*p* < 0.0001), NCA at 1.97 *μ*M (% inhibition: 69.35 ± 6.61) (*p* < 0.0001), CCA at 0.35 *μ*M (% inhibition: 68.85 ± 9.85) (*p* < 0.0001), and CGA at 0.56 *μ*M (% inhibition: 76.67 ± 1.63) (*p* < 0.0001), as well as by CAF at 0.52 *μ*M (% inhibition: 71.85 ± 4.92) (*p* < 0.0001) and RUT at 0.16 *μ*M (% inhibition: 63.76 ± 5.51) (*p* < 0.0001). The concentration of each compound used in these experiments represents the amount of each one in the more active extract (IA) at the lower concentration tested (3 *μ*g/mL). Moreover, when we compare the potency of each one to inhibit the NOx production, it is possible to verify that all of them were more potent than the reference drug used, particularly theobromine which was 200x more potent than dexamethasone (Table 3).

**Figure 8:**
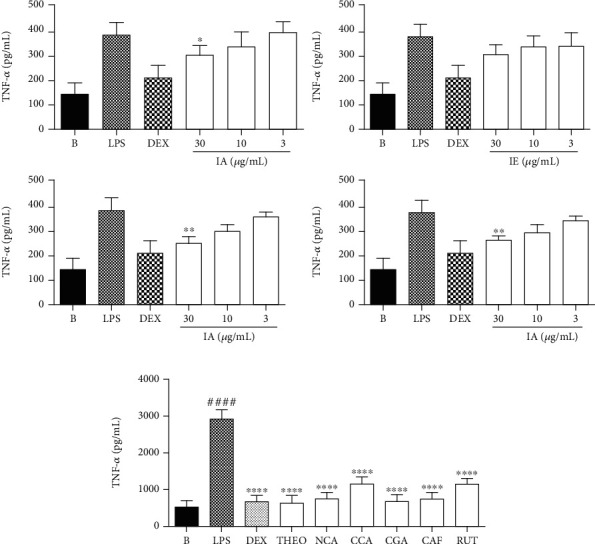
Effects of chemical marker compounds on TNF-*α* secretion levels in LPS-treated RAW 264.7 macrophages. B: untreated cells; LPS: cells treated with only lipopolysaccharide (1 *μ*g/mL); DEX: cells pretreated with dexamethasone (7 *μ*M) before LPS administration. Results were expressed as mean ± SEM; *n* = 3. (a) Extracts: ^∗^*p* < 0.05 vs. group B or ^∗∗^*p* < 0.005. IA: aqueous infusion; IE: ethanolic infusion; TA: aqueous turboextraction; TE: ethanolic turboextraction. (b) Compounds: ^####^*p* < 0.0001 vs. group LPS; ^∗∗∗∗^*p* < 0.01 vs. group B. THEO: theobromine; NCA: neochlorogenic acid; CCA: cryptochlorogenic acid; CGA: chlorogenic acid; CAF: caffeine; RUT: rutin.

The secretion of TNF-*α* assay was also significantly inhibited by the IA, TA, and TE extracts at 30 *μ*g/mL (Figure 8(a)) as well as by all chemical markers present in IA extract (Figure 8(b)). Furthermore, TNF-*α* secretion was significantly decreased by IA at doses of 30 *μ*g·mL^−1^ (% inhibition: 83.33 ± 4.01) (*p* < 0.05), 10 *μ*g·mL^−1^ (% inhibition: 75.00 ± 1.86) (*p* > 0.05), and 3 *μ*g·mL^−1^ (% inhibition: 107.89 ± 4.55) (*p* > 0.05); by IE at doses of 30 *μ*g·mL^−1^ (% inhibition: 86.84 ± 4.55) (*p* > 0.05), 10 *μ*g·mL^−1^ (% inhibition: 88.15 ± 5.58) (*p* > 0.05), and 3 *μ*g·mL^−1^ (% inhibition: 78.94 ± 7.44) (*p* > 0.05); by TA at doses of 30 *μ*g·mL^−1^ (% inhibition: 63.15 ± 2.63) (*p* < 0.005), 10 *μ*g·mL^−1^ (% inhibition: 76.31 ± 2.63) (*p* > 0.05), and 3 *μ*g·mL^−1^ (% inhibition: 92.54 ± 2.00) (*p* > 0.05); and by TE at doses of 30 *μ*g·mL^−1^ (% inhibition: 67.10 ± 1.86) (*p* < 0.005), 10 *μ*g·mL^−1^ (% inhibition: 75.43 ± 3.03) (*p* > 0.05), and 3 *μ*g·mL^−1^ (% inhibition: 93.42 ± 2.27) (*p* > 0.05). The LPS increase the TNF-*α* secretion levels, while the dexamethasone (DEX), at 7 *μ*M, reduced the levels of this proinflammatory mediator (% inhibition: 23.92 ± 5.05) (*p* < 0.0001). Furthermore, TNF-*α* secretion was significantly decreased by THEO tested at 0.033 *μ*M (% inhibition: 22.15 ± 6.49) (*p* < 0.0001), NCA at 1.97 *μ*M (% inhibition: 27.46 ± 3.98) (*p* < 0.0001), CCA at 0.35 *μ*M (% inhibition: 39.76 ± 5.73) (*p* < 0.0001), and CGA at 0.56 *μ*M (% inhibition: 23.58 ± 5.79) (*p* < 0.0001), as well as by CAF at 0.52 *μ*M (% inhibition: 26.45 ± 5.34) (*p* < 0.0001) and RUT at 0.16 *μ*M (% inhibition: 40.18 ± 3.70) (*p* < 0.0001). The concentration of each compound used in these experiments represents the amounts of each one in the IA at the lower concentration tested (3 *μ*g/mL).

## 4. Discussion

The chemical profile of *I. paraguariensis* extracts was investigated by FC assay and UPLC/DAD. TPC analysis showed that IA, IE, and TE extracts possess high amounts of phenolic compounds. Also, UPLC/DAD showed THEO, NCA, CCA, CGA, CAF, and RUT as the major compounds while UPLC/MS analysis confirmed these compounds and the presence of quinic acid, citric acid, caffeoylglucose, feruloylquinic acid, quercetin-glycoside, and kaempferol-rhamnoglucoside. All these data are in agreement with the literature data [18, 19, 20, 31, 36]. Considering the high amounts of phenolic compounds detected in *I. paraguariensis* preparations, the antioxidant activity of these extracts and its chemical markers was also investigated by the DPPH assay. This is a free radical scavenging method that offers the first approach for evaluating the antioxidant potential of a compound, extract, or other biological material. This is the simplest method, wherein the prospective compound or extract is mixed with DPPH solution and absorbance is recorded after a predefined period, based on the measurement of the scavenging capacity of antioxidants towards it [38]. The extracts exhibited strong free radical scavenging, since IA showed EC_50_ at 54.4 ± 5.1 *μ*g/mL.

Phenolic compounds have been linked to favorable impacts in healthcare, particularly due to their antioxidant activity [39]. Studies have suggested an inverse correlation between fruit and vegetable consumption and serum levels of inflammatory markers [40]. In addition, it is also evidenced that oxidative stress plays a pathogenic role in chronic inflammatory diseases [41], since the oxygen radical absorbance capacity assay showed that phenolic compounds present in the extracts are a strong antioxidant agent, and more importantly, these metabolites also inhibit cytokines such as TNF-*α* and IL-6 [42]. Therefore, a considerable number of studies have investigated the antioxidant [20, 43] and anti-inflammatory properties of *I. paraguariensis* [18–20].

The anti-inflammatory activity of *I. paraguariensis* was previously described [18–20]. Schinella et al. [20] showed that oral administration of the aqueous mate extract significantly reduced the carrageenan-induced edema, an effect that was accompanied by a 43% and 53% reduction of the expression of cyclooxygenase-2 and inducible nitric oxide synthase, respectively. Luz et al. [19] showed that a hydroethanolic extract of *I. paraguariensis* leaves was able to reduce leukocyte migration, exudate concentration, MPO and ADA activities, and NOx levels. The aqueous extract was also beneficial in adjuvant-induced arthritic rats by diminishing both inflammation and oxidative stress [18]. Our study therefore reinforces the anti-inflammatory activity of standardized extract of *I. paraguariensis* and demonstrates the strong activity of IA on NOx secretion, especially at the lowest dose (3 *μ*g/mL). This anti-inflammatory activity detected for *I. paraguariensis* extracts can be explained, in part, by the presence of phenolic compounds in the extracts.

In view of previous reports that describe the potential anti-inflammatory properties of hydroxycinnamic acid derivatives [44], we also investigated the effects of chemical markers on NOx secretion. The results reinforce the potential of chlorogenic acid and its structural analogues (NCA; CCA) as anti-inflammatory agents. CGA is one of the most widely available polyphenols among consumers because it exists in most abundant quantities in different foods [45].

It is also important to highlight that theobromine also showed strong inhibition of NOx secretion. This compound is not used in clinical care, but pentoxifylline, a synthetic theobromine derivative, is an immunological agent that is sometimes used to treat pediatric septic shock. Pentoxifylline acts by inhibiting erythrocyte phosphodiesterase, which increases expression of the anti-inflammatory protein adenosine monophosphate protein kinase (AMPK), suppressing neutrophils and proinflammatory cytokines and probably preventing chronic inflammation [46, 47].

Nitric oxide is an important signaling molecule, and it is also highly reactive and diffusible. Therefore, it is important to have a strict control and regulation of production under physiologic conditions [48]. This molecule plays many important roles in the immune system, as well as on inflammation conditions. It is produced in high amounts from specialized cells of the immune system, mainly for cells of the innate immune system as macrophages.

TNF-*α* also plays as an important inductor of inflammation in several pathologies. TNF-*α* is one of the most important inflammatory cytokines that controls different types of cell functions. This inflammatory cytokine is produced by macrophages/monocytes during acute inflammation and is responsible for a diverse range of signaling events within cells, leading to necrosis or apoptosis [49]. Therefore, it is evident that the suppression of TNF-*α* could be beneficial in different inflammatory diseases.

The effect of dietary habit on TNF-*α* represents an important subject with significant clinical implications, since several researches showed that phenolic-rich food intake was significantly and inversely related to TNF-*α* and IL-6 secretion [40]. Although the precise mechanisms of this anti-inflammatory activity are not fully elucidated, it is has been hypothesized that phenolic compounds exert anti-inflammatory activity by inhibiting the synthesis of proinflammatory mediators, modification of eicosanoid synthesis, inhibition of activated immune cells, or inhibition of nitric oxide synthase and cyclooxygenase-2 via its inhibitory effects on nuclear factor NF-*κβ* [50].

According to previous study described by Correa et al. [18], our data also showed that chlorogenic acid and its structural analogues were effective to suppress the TNF-*α* secretion levels in LPS-treated RAW 264.7 macrophages. Furthermore, similar potential was detected for THEO, CAF, and RUT, since all these compounds were able to suppress the TNF-*α* secretion as DEX.

NOx, TNF-*α*, and reactive oxygen species (ROS) are responsible for the nuclear transcription factor kappa (NF-*κ*B) activation, which leads to the upregulation of many proinflammatory genes [51]. Our results showed that IA and its major chemical markers possess a strong potential to suppress NOx, TNF-*α*, and ROS production, suggesting that IA extracts and the related major compounds present in a plant extract may work based on several different mechanisms synergistically, resulting in moderating the immune system.

## 5. Conclusion

According to the results described herein, the extract prepared by infusion showed the best results in terms of anti-inflammatory and antioxidant activities and total phenolic content. In addition, all major chemical markers present in the IA extract showed a strong anti-NOx activity and suppress the TNF-*α* secretion. Among these chemical markers, theobromine was highlighted, since in comparison with reference anti-inflammatory drug (DEX) it showed the same effect but at concentration 200x lower.

## Figures and Tables

**Table 1:** Content of the major compounds of *I. paraguariensis* extracts by LC-UV (mg·g^−1^). Different letters indicate significant difference content in the same compound among different extraction procedures (ANOVA, followed by Tukey test; *p* < 0.05).

Compound	IA	IE	TA	TE
(THEO)	2.12 ± 0.06^a^	2.27 ± 0.08^a^	1.74 ± 0.02^b^	2.07 ± 0.01^a^
(NCA)	273.54 ± 3.16^a^	273.29 ± 1.98^a^	243.58 ± 4.21^b^	276.76 ± 3.20^a^
(CCA)	40.84 ± 0.49^a^	30.29 ± 0.4^b^	23.88 ± 0.17^c^	27.82 ± 0.37^d^
(CGA)	56.83 ± 0.58^a^	55.82 ± 1.73^a^	52.00 ± 0.73^b^	59.07 ± 0.34^a^
(CAF)	22.55 ± 0.23^a^	20.96 ± 0.22^b^	17.64 ± 0.30^c^	23.18 ± 0.16^a^
(RUT)	39.28 ± 0.28^a^	42.07 ± 0.90^a^	38.81 ± 0.24^b^	35.98 ± 1.11^c^

Note: IA: aqueous infusion; IE: hydroethanolic infusion; TA: aqueous turboextraction; TE: hydroethanolic turboextraction; THEO: theobromine; NCA: neochlorogenic acid; CCA: cryptochlorogenic acid; CGA: chlorogenic acid; CAF: caffeine; RUT: rutin.

**Table 2:** Characterization of the major phytoconstituents of *Ilex paraguariensis* by LC-MS.

No.	Mode	RT (min)	Expt. *m*/*z*	Fragments *m*/*z*	Molecular formula	PPM	Common names
1	(−)	2.70	191.0550	85.0295 127.0399 173.0990	C_7_H_12_O_6_	-3.1	Quinic acid^a^^∗∗^
2	(−)	3.22	191.0183	85.0266 87.0088 111.0080 173.0101	C_6_H_8_O_7_	-4.7	Citric acid^a^^∗^
3	(+)	5.48	181.0734	108.0562 122.0582 138.0664 163.0607	C_7_H_8_N_4_O_2_	4.4	Theobromine^b^
4	(−)	5.58	353.0873	135.0445 179.0343 191.0550	C_16_H_18_O_9_	0.0	5-O-caffeoylquinic acid^b^
5	(−)	5.81	341.0887	161.0267 179.0349 203.0353 221.0402 323.0784	C_15_H_18_O_9_	4.1	Caffeoylglucose^a^^∗∗^
6	(−)	6.07	341.0887	161.0267 179.0349 203.0353 221.0402 323.0784	C_15_H_18_O_9_	4.1	Caffeoylglucose^a^^∗∗^
7	(−)	6.34	353.0873	135.0445 173.0451 179.0343 191.0550	C_16_H_18_O_9_	0.0	4-O-Caffeoylquinic acid^b^
8	(−)	6.34	353.0873	135.0445 173.0451 179.0343 191.0550	C_16_H_18_O_9_	0.0	3-O-Caffeoylquinic acid^b^
9	(+)	6.67	195.0886	110.0723 123.0430 138.0664	C_8_H_10_N_4_O_2_	2.1	Caffeine^b^
10	(−)	6.77	367.1033	135.0445 173.0456 179.0343	C_17_H_20_O_9_	-2.5	Feruloylquinic acid^∗∗^
11	(−)	7.03	515.1195	135.0445 173.0451 179.0343 191.0550 353.0873	C_25_H_24_O_12_	1.0	4,5-di-*O*-Caffeoylquinic acid^a^^∗^
12	(−)	7.1	515.1195	135.0445 173.0451 179.0343 191.0550 353.0873	C_25_H_24_O_12_	1.0	3,5-di-*O*-Caffeoylquinic acid^a^^∗^
13	(−)	7.4	515.1195	135.0445 173.0451 179.0343 191.0550 353.0873	C_25_H_24_O_12_	1.0	3,4-di-*O*-Caffeoylquinic acid^a^^∗^
14	(−)	7.5	609.1490	301.0358 463.0884	C_27_H_30_O_16_	—	Rutin^b^
15	(−)	7.6	463.0884	301.0358	C_21_H_20_O_12_	1.5	Quercetin-glycoside^∗∗^
16	(−)	7.9	593.1522	285.0425	C_27_H_30_O_16_	2.7	Kaempferol-rhamnoglucoside^∗∗^

^a^Identified by data in the literature. ^b^Identified with authentic standards. ^∗^Sokeng et al. [37]. ^∗∗^Mateos et al. [36].

**Table 3:** Effects of compounds/chemical markers at concentration present in the IA extract (at a dose of 3 *μ*g/mL) on NOx concentrations in the RAW 264.7 macrophages induced by LPS.

Chemical markers	Chemical marker concentration (*μ*M)	Potency^∗^
(THEO)	0.033	212.12
(NCA)	1.97	3.55
(CCA)	0.353	19.83
(CGA)	0.565	12.39
(CAF)	0.515	13.59
(RUT)	0.164	42.68

THEO: theobromine; NCA: neochlorogenic acid; CCA: cryptochlorogenic acid; CGA: chlorogenic acid; CAF: caffeine; RUT: rutin; DEX: dexamethasone (7 *μ*M). The potency of each chemical marker is represented by a ratio among dexamethasone (at 7 *μ*M) and the concentration of each compound in the IA extract (at a dose of 3 *μ*g/mL; % NOx inhibition: 42.86 ± 6.83) (*p* < 0.0001).

## Data Availability

The data that support the findings of this study are available from the corresponding author.
